# The Formation of Biofilm and Bacteriology in Otitis Media with Effusion in Children: A Prospective Cross-Sectional Study

**DOI:** 10.3390/ijerph18073555

**Published:** 2021-03-30

**Authors:** Artur Niedzielski, Lechosław Paweł Chmielik, Tomasz Stankiewicz

**Affiliations:** 1 Clinic of Pediatric Otolaryngology, Center of Postgraduate Medical Education (CMKP), 01-813 Warsaw, Poland; l.p.chmielik@chmielik.pl; 2 Independent Otoneurological Laboratory, Medical University of Lublin, 20-093 Lublin, Poland; tomaszstankiewicz@umlub.pl

**Keywords:** otitis media with effusion, child, biofilms

## Abstract

Background: Otitis media with effusion (OME) can cause serious complications such as hearing impairment or development delays. The aim of the study was to assess the microbiological profile of organisms responsible for OME and to determine if a biofilm formation can be observed. Methods: Ninety-nine samples from 76 patients aged from 6 months to 12 years were collected for microbiological and molecular studies. Results: In microbiological studies, pathogenic bacteria *Haemophilus influenzae* (38.89%), *Streptococcus pneumoniae* (33.33%), and *Staphylococcus aureus MSSA* (27.78%), as well as opportunistic bacteria *Staphylococcus* spp. (74.14%), *Diphtheroids* (20.69%), *Streptococcus viridans* (3.45%), and *Neisseria* spp. (1.72%) were found. The average degree of hearing loss in the group of children with positive bacterial culture was 35.9 dB, while in the group with negative bacterial culture it was 25.9 dB (*p* = 0.0008). The type of cultured bacteria had a significant impact on the degree of hearing impairment in children (*p* = 0.0192). In total, 37.5% of *Staphylococcus* spp. strains were able to form biofilm. Conclusions: *Staphylococcus* spp. in OME may form biofilms, which can explain the chronic character of the disease. Pathogenic and opportunistic bacteria may be involved in the etiopathogenesis of OME. The degree of hearing loss was significantly higher in patients from which the positive bacterial cultures were obtained.

## 1. Introduction

Otitis media with effusion (OME) is a chronic inflammatory condition of the middle ear without general symptoms of acute infection. The disease is characterized by the presence of fluid in the tympanic cavity and conductive hearing loss. OME is one of the most common diseases in childhood. Two-thirds of children have had at least one episode of OME by the age of 3 years. One-third of them will have the attack without notice; therefore, it is called “silent” otitis media and can impair their hearing secretly [1]. It is also the most common cause of hearing loss in the pediatric population, which may adversely affect the development of speech as well as linguistic and cognitive abilities [2].

The pathogenesis of the disease is not fully understood and is most likely multifactorial. The development of recurrent and chronic ear infections is influenced by individual and environmental factors [3]. The individual risk factors for exudative otitis include: (1) anatomical and functional dysfunction of the eustachian tube [4]; (2) genetic predisposition [5]; (3) male gender [6]; (4) recurrent infections of the upper respiratory tract [7]; (5) episode of acute otitis media in the first 6 months of life [8]; (6) developmental defects in the craniofacial region, especially cleft palate and abnormal structure of the mastoid process with impaired pneumatization [9,10]; (7) the overgrowth of the Waldeyer’s tonsillar ring [11]; (8) gastroesophageal reflux disease [12]; (9) immunological disorders [13]; and (10) allergy [14]. The factors of increased risk of OME in relation to environmental factors [15] play the most important role: (1) exposure to tobacco smoke; (2) attendance at a day care center (nursery, kindergarten); (3) poor socioeconomic status; and (4) autumn and winter seasons [15].

In most exudates in acute otitis media, *Haemophilus influenzae, Streptococcus pneumoniae*, and *Moraxella catarrhalis* are found. On the other hand, cultures of exudate in OME indicate no bacteria. Moreover, exudate is resistant to antibiotic treatment and susceptible to many inflammatory mediators, which led to the concept of its sterility. However, polymerase chain reaction (PCR) improved the sensitivity of bacterial detection in middle ear infections and is useful for the detection of pathogens that are slowly growing, difficult to culture, or hazardous to handle in a diagnostic lab [16]. Reason for a better detection of bacteria with PCR may be due to a small number of microorganisms that do not reach the limit of detection by direct culturing [17,18,19,20,21,22,23,24,25,26].

Using PCR technique, in 20–50% of the middle ear exudates, the *Alloiococcus otitidis* bacterium was detected. It has been found that this bacterium has a high immunostimulatory capacity and promotes colonization of the middle ear space [27,28].

It has long been known that 70% of the cultures are sterile in OME. Numerous reports indicate the lack of effects of antibiotic therapy in OME, indicating biofilm as the causative agent of the chronic nature of the disease [29].

Bacteria in nature often exist as sessile communities called biofilms [30]. These communities develop structures that are morphologically and physiologically differentiated from free-living bacteria [30] and more resistant to external factors. The concept of disease based on the existence of bacterial biofilm [31,32,33,34,35] explains why in a chronic bacterial infection it may be difficult to obtain positive cultures and explains the relative failure of treatment of OME with antibacterial drugs [30]. However pharmacokinetic drug penetration studies consistently indicate that the bactericidal concentration of the drug is easily achieved in the ear, and studies on planktonic bacteria indicate their sensitivity in vitro to antibacterial agents. The mucosal biofilm hypothesis also explains the observation that the most effective treatment of OME is tympanostomy with ear drainage. The poorly ventilated middle ear is an excellent environment for the formation of a bacterial mucosal biofilm. Ventilation tube surgery is an effective procedure for the following reasons: (1) restoration of middle ear ventilation increases oxygen concentration in this area, potentially changing the biofilm phenotype, (2) mechanical suction of exudate after tympanic membrane cutting interrupts continuity, cleanses, and reduces the mass of biofilm, and (3) restoration of ventilation facilitates reconstructing the host’s defensive mechanisms in the mucosa of the middle ear. These changes lead to purification of the biofilm and remission of exudate [21,36,37].

The aim of the work was to evaluate the microorganisms responsible for otitis media with effusion development and their ability to form biofilms.

## 2. Materials and Methods

Our study included 76 patients admitted for surgical treatment of otitis media with effusion. Among 76 children participating in the study, 44 were boys (57.9%) and 32 were girls (42.1%), and their age ranged from 6 months to 12 years. All patients underwent basic laryngological and hearing evaluation according to age (impedance audiometry, otoacoustic emissions, pure-tone threshold audiometry). Impedance audiometry [38] was performed with the Madsen Zodiak 901 clinical tympanometer (GN Otometrics, Taastrup, Denmark), and otoacoustic emissions [39] were tested by the OtoRead™ Otoacoustic Emission Test Instrument (Interacoustics, Middelfart, Denmark). Air and bone conduction pure-tone auditory [40] threshold measurement was performed in cooperating patients using the Madsen Orbiter 922-2 clinical audiometer (GN Otometrics, Denmark). Based on the hearing evaluation, patients underwent either unilateral or bilateral tympanotomy. During the surgery we collected 99 samples from the middle ear space. From patients with bilateral OME, due to the small amount of the exudate, we collected two samples from the same patient in order to perform all needed examinations. Each sample was divided into two parts, one of which was used for microbiological culture and the other for molecular biology.

After cleaning and disinfection with a 70% spirit solution of the external auditory canal and the eardrum, tympanic membrane was cut in the posterior-lower quadrant using an operating microscope. The material was collected under pressure using a Polymed Mucus Extractor disposable sterile collection set for Poly Pedicure Ltd. (Ballabhgarh, India).

Middle ear aspirates were inoculated onto Columbia medium with 5% sheep blood, Chapman medium, MacConkey agar, chocolate agar with bacitracin, and Sabouraud agar. Then media were incubated at 37 °C for 24 h under aerobic conditions. Only chocolate agar with bacitracin was incubated under microaerophilic conditions 5–10% CO_2_ using an anaerostat, and Sabouaraud agar was incubated at 30 °C for up to 5 days. After incubation, macroscopic assessment of the growth of bacteria and fungi on the media was made and colony morphology was determined (i.e., shape, size, surface, color, transparency).

To identify species of the bacteria of the Staphylococcus genus catalase test [41], the Slidex Staph-Kit agglutination test (bioMerieux, Marcy-l’Étoile, France) [42], coagulase free test [43], and the API Staph system tests (bioMerieux) [44] were performed.

To detect bacteria of the *Streptococcus* genus, the following tests we performed: microscreen Strep latex confirmation assay (Lab M, Neogen, Heywood, UK), optochin resistance test [45], and the API 20 Strep system tests (bioMerieux) [46].

Identification of *Haemophilus* species was made using X and V growth factor requirement tests. Depending on the species, bacteria need separate growth factors X (hemin or hematine) and V (NAD or NADP) to develop. Firstly, the plates were brought to room temperature and then the pure culture of the *Haemophilus* strain was suspended in a sterile 0.9% NaCl solution to obtain a suspension with a density of about 0.5 McFarland. After the suspension was inoculated with a sterile cotton swab onto Muller–Hinton agar standardized according to National Committee for Clinical Laboratory Standards (NCCLS) recommendations, the diagnostic discs BVX, BV, and BX (B-bacitracin, factor V, factor X) were plated at a distance of about 15–20 mm apart from each other. Then the material was incubated at 35 °C for 18–24 h in an atmosphere of 5–7% CO_2_.

Molecular microbial analysis was performed based on the polymerase chain reaction (PCR), which consists of the following stages: isolation of genomic DNA from cells present in the exudate and from coagulase positive staphylococci grown from these fluids, amplification of a specific fragment of the isolated genetic material of the microorganism, and detection of the amplified product. Genomic DNA was isolated from exudate and *Staphylococcus sp.* using the manufacturers protocol. The amount and purity of the DNA was checked using an Eppendorf BioPhotometer (Eppendorf AG, Hamburg, Germany). The largest amount of genomic DNA obtainable using the test described above was 60 μg. The purity of the sample was determined as the ratio of absorbance at 260 nm and 280 nm and for pure DNA (the index A260/280 is: 1.7–1.9). Measurements were made in disposable cuvettes with the Eppendorf BioPhotometer measurement instructions.

For samples with negative culture results (*n* = 61), a PCR reaction was performed. The DNA fragment encoding the bacterial 16s rRNA subunit in the nested-PCR system was amplified. The test was based on two amplification reactions: outer (Table 1), in which the resulting product was 740 base pairs; and a nested reaction (Table 2), in which the resulting product was 290 base pairs. 

The resulting PCR product of 740 base pairs could or could not be detected on an agarose gel stained with ethidium bromide. This PCR product, however, was a template for the nested reaction in which primers complementary to the sites within the 740 bp product were used. Thus, a product of 290 base pairs was formed, which could be visible in the agarose gel in the form of a clear band for a positive sample. The nested reaction was carried out in the case of a negative result of the outer reaction. 

The composition of the outer PCR reaction mixture for one sample included: 39.0 μL master mix PCR-out, 5.0 μL dNTPs mixture, 5.0 μL DNA, and 1.0 μL of Delta2 polymerase. The nested PCR reaction mixture for one sample consisted of: 39.0 μL master PCR-In, 5.0 μL dNTPs mixture, 5.0 μL of the PCR product obtained in the initial amplification, and 1.0 μL of Delta2 polymerase. Primers used in outer and nested PCR reactions were described by Gok et al. [47]. The amplified product was detected with 2% agarose gel electrophoresis [48]. In our study we used 10 μL of amplification product and 3 μL of dye (bromophenol blue). To determine the position of the amplification reaction product, a DNA size marker was used: ΦX174 DNA/BsuRI (MBI, Fermentas, Lithuania). For each PCR reaction positive and negative controls were performed.

The ability to form biofilm by coagulase-negative staphylococci (CNS) strains was tested by using Congo Red Agar method (CRA), Tissue Culture Plate (TCP) and determination of the presence of the *ica* operon genes in CNS strains.

Congo Red Agar method (CRA), prepared as described by Freemen and colleagues in 1989, was used to determine if coagulase-negative staphylococci can form biofilm. Firstly, plates with medium were inoculated and incubated to obtain single colonies. After 24 h at 37 °C, positive strains appeared as black colonies with a dry, crystalline consistency, while the polysaccharide negative strains remained red.

Tissue Culture Plate (TCP) is considered the gold standard phenotypic method of biofilm detection. In this method, bacterial adherence is measured spectrophotometrically [49]. In our study, a suspension of bacteria with a density of 0.5 MF (Mc Farland) was incubated at 37 °C for 24 h under aerobic conditions, then diluted in 1: 100 TSB buffer. A portion of 100 μL of the suspension of each strain (3 replicates for one strain) was applied onto the microplate and incubated at 37 °C for 24 h in aerobic conditions. The plate was rinsed 3 times with TSB buffer and then the plate was stained with 0.1% crystal violet for 15 min. After washing the dye, 100 μL of absolute alcohol was added to each well. The absorbance reading was performed in an ELISA reader at 570 nm. As a negative control, we used a *Staphylococcus epidermidis* ATCC 12228 reference strain, which does not produce a biofilm. The positive result was the absorbance greater than twice the mean absorbance value read for the negative control.

Determination of the presence of the intercellular adhesion (*ica)* operon genes in cultured coagulase-resistant staphylococci strains was made as follows. Genomic DNA was isolated from exudate and *Staphylococcus sp.* using the manufacturer’s protocol. In order to identify the presence of *ica* operon genes in coagulase-negative staphylococci, PCR reactions were performed using primers for the *icaA*, *icaB*, *icaC*, and *icaD* genes. The primer sequences for each of the *ica* operon genes, the amplification conditions for each pair of primers, and the size of the amplification products are shown in the following studies of Ziebuhr [50] and de Silva [51] (Table 3).

The composition of the reaction mixture for one 50 μL sample included 5 μL amplification buffer, 2.5 μL primer 1, 2.5 μL primer 2, 3.0 μL MgCl2, 1 μL dNTPs, 0.5 μL Taq Polymerase (5 U/μL), 5 μL template DNA, 30.5 μL H2O. The *icaA*, *icaB*, *icaC*, and *icaD* gene amplification products were detected with 2% agarose gel electrophoresis [48]. The positive result was manifested by the presence of a band of appropriate size in each gel for each gene. To determine the position of the amplification product, a DNA size marker was used: ΦX174 DNA/BsuRI (MBI, Fermentas, Lithuania) [51,52].

Data analysis was performed using SPSS 13 (SPSS Inc., Chicago, Illinois, United States of America). The independent samples *t*-test was used to compare differences between the groups. A *p* ≤ 0.05 was considered statistically significant.

The study was conducted according to the guidelines of the Declaration of Helsinki and approved by the Bioethical Committee of Medical University of Lublin.

## 3. Results

### 3.1. Results of Microbiological Tests

Out of 99 samples, positive cultures were found in 38 samples (38.38%), whereas no bacteria were grown in the remaining 61 samples (61.62%). The average degree of hearing loss in the group of children with positive bacterial culture was 35.9 dB, while in the group with negative bacterial culture it was 25.9 dB (*p* = 0.0008) (Table 4).

PCR reaction confirmed the presence of bacteria in the exudate collected from the middle ear of patients with OME. In the outer reaction, bacterial DNA was confirmed in 19 cases (19.19%), while in the nested reaction, it was found in 42 cases (42.42%). 

Opportunistic bacteria were detected much more frequently (76.32%) than pathogenic (23.68%) (Table 5). 

Among the pathogenic bacteria *Haemophilus influenzae* was most frequently isolated (38.89%) in our study. Other cultured pathogens were identified as *Streptococcus pneumoniae* (33.33%) and *Staphylococcus aureus MSSA* (27.78%) (Table 6). 

Amid the opportunistic bacteria, *Staphylococus* spp. (74.14%) and *Diphtheroids* (20.69%) predominated in the exudate collected from the middle ear space. In addition, *Streptococcus viridans* (3.45%) and *Neisseria* spp. (1.72%) were also found (Table 7).

Based on our study, the type of cultured bacteria has a significant influence on the degree of hearing loss in children (*p* = 0.0192). The average degree of hearing loss in the group of children with pathogenic bacteria found in their exudate was higher (32.3 dB) than in the group with opportunistic bacteria (27.5 dB) (Table 8).

### 3.2. Bacterial Biofilm Formation Results

Due to the large amount of *Staphylococcus* spp. bacteria in the culture (74.14%), an attempt was made to analyze the phenotypic and genotypic ability of *Staphylococcus* spp. strains to form biofilm. Out of all *Staphylococcus* spp. isolates, the Api Staph study showed that the most commonly isolated bacteria were *S. epidermidis* (50%). Next predominating organisms were *S. aureus* (18.75%) and *S. sciuri* (12.5%). The remaining strains were identified as: *S. capitis* (6.25%), *S. caprae* (6.25%), and *S. verneri* (6.25%) (Table 9).

Out of all *Staphylococcuss* spp. isolates, 37.5% showed a phenotypic ability to form biofilm, as confirmed by CRA and TCP methods. In addition, 12.5% of *S. epidermidis* strains were *icaA*, *icaB*, *icaC*, and *icaD* positive and 6.25% of *S. epidermidis* strains were only *icaA* and *icaD* positive in PCR. However, 18.75% of *S. epidermidis* were also positive in the TCP and CRA methods, which confirms their biofilm formation potential.

Lastly, 80% of *S. aureus* strains, detected by PCR analysis as *icaA* or *icaB* positive, showed a biofilm negative phenotype in the TCP and CRA methods.

## 4. Discussion

Research by Bluestone et al. [17] showed that the most common pathogens in ear infections were *Streptoccocus pneumoniae, Haemophilus influenzae*, and *Moraxella catarrhalis*. However, the authors found a different percentage of these bacteria in acute otitis media (AOM) and OME. In AOM, the most common bacterium was *Streptococcus pneumoniae*, isolated in 35% of cases, whereas in OME it was found in only 7% of cases. Contrarily, in OME the most frequently isolated bacterium was *Haemophilus influenzae* (15% of exudates) and the second most frequent bacterium was *Moraxella catarrhalis*, cultured in 10% exudates. This study was conducted on the exudate collected from 4589 ears and it is often a model for other researchers. Bluestone did not obtain bacterial growth in 30% of samples, whereas in our study no bacteria were cultured in 61.62% of the samples. Other bacteria considered to be non-pathogenic constituted 45% in Bluestone’s research, while in our study opportunistic bacteria were found in 76.32% of positive culture samples. In addition to the three major pathogens, Bluestone et al. identified *Streptococcus aureus* 2%, *Streptococci group A* 1%, *Streptococci alpha* 3%, and *Pseudomonas aeruginosa* [53] 2%. In our study, out of 99 samples collected from middle ear space, *Haemophilus influenzae* was detected in 3.53% of cases, *Streptococcus pneumoniae* in 3% of samples, *Staphyloccocus aureus MSSA* (Methicillin-sensitive *Staphylococcus aureus*) in 2.52% of cases, and *Streptoccocus viridans* in 1% of samples. The most commonly cultured microorganism in our study was *Staphylococcus* spp. (21.71%), and no growth of *Moraxella catarrhalis* was found in the culture. Comparable results were obtained by Park et al. [52], who confirmed the presence of *Haemophilus influenzae* in 7.9% of cases and *Streptococcus pneumonae* in 1.4% of cases, while *Moraxella catarrhalis* was not grown. On the other hand, Poetker et al. [22] reported that the most frequently isolated *Staphylococcus species NOS* (not otherwise specified) was identified in 38 samples from 148 (25.7%). The decrease in the percentage of major pathogens compared with Bluestone’s studies, especially reduction of the amount of *Streptococcus pneumoniae*, may be associated with the usage of pneumococcal vaccine. Similar observations are also noted by other authors [54,55].

The positive identification of bacteria by PCR suggests a bacterial etiology for OME. Palmu et al. identified *S. pneumoniae* in 47.1% of middle ear effusion using PCR, compared with 27.3% using standard cultures [56]. In studies of Park et al. [52], bacteria in culture were detected in 14% of cases, while using PCR techniques, bacterial DNA was isolated in 36.7% [52]. Similar results were obtained in research by Choi et al. [57]. In our study, bacteria in culture were detected in 38.38% of cases, whereas by PCR in 61.62% of samples, using outer and nested technique (19.19% and 42.42%, respectively). The authors discuss such a low bacterial detection rate in cultures, explaining this phenomenon with antibiotic therapy before ear drainage, the presence of secretory immunoglobulins, and lysozyme in the middle ear secretion inhibiting bacterial growth, as well as the presence of bacteria in the middle ear in the form of biofilms [52,58].

OME can cause hearing impairment, and in our study the hearing loss varied in the range of 25–40 dB. What is interesting is that the degree of hearing loss was significantly higher in patients from which positive bacterial cultures were obtained. The difference in the audiometric test was on average 10 dB and was statistically significant (*p* = 0.0008). Thus, in the ears with a positive culture, hearing loss was 35.1 dB on average. A positive correlation was also observed between the degree of hearing loss and the presence of pathogenic bacteria as compared with non-pathogenic bacteria. In the case of pathogenic bacteria, the degree of hearing loss was greater (*p* = 0.0192). To the best of our knowledge, no study has been published showing the correlation between the degree of hearing loss and the type of bacteria found in exudate. However, experimental studies could provide some possible reasons why the degree of hearing loss differs between positive and negative cultures. Stenqvist et al. studied electrophysiological changes in the albino rat following instillation of *Pseudomonas aeruginosa* exotoxin A into the middle ear cavity [59]. They found that *Pseudomonas aeruginosa* exotoxin A causes middle ear inflammation, facilitating penetration to the inner ear and that this toxin also reversibly affects cochlear function—*Pseudomonas aeruginosa* exotoxin A raised the ABR threshold over the whole frequency range by 5–25 dB [59], which can explain why in our study the degree of hearing loss was significantly greater in the children in which bacteria were detected.

*S. epidermidis* is the main organism isolated from foreign material related infections (FMRI), such as infected prosthetic joints, central venous catheters, cerebrospinal fluid shunts, intracardiac devices, artificial heart valves, and vascular grafts [60]. In our study, *S. epidermis* had proven to be the most common microorganism of *Staphylococcus species* to form biofilm in OME. Daniel et al. examined bacterial involvement in OME using confocal laser scanning microscopy (CLSM) and bacterial viability stain [61]. They noticed that among the CLSM-positive samples, 49.0% contained biofilms, and the most common pathogen to form biofilm was *Psudomonas* spp. (4.8%). They also found that coagulase-negative *staphylococci* (CoNS) dominated in the culture (12.9%), and out of all CoNS strains, *S. epidermidis* (3.2%) and *S. lugdunensis* (3.2%) were the most common, similar to our findings. However, among CoNS strains, 25% were able to form biofilm based on their findings [61]. In our study, 37.5% of all *Staphylococcuss* spp. strains showed a phenotypic ability to form biofilm.

It is likely that freely drifting, planktonic bacteria are far less common than those associated with biofilms. The biofilm explains the presence of metabolically active bacteria as well as bacterial endotoxins, despite negative cultures from the middle ear. 

The OME etiology model is a chronic middle ear effusion as a result of biofilm formation from pathogenic bacteria on the middle ear mucosa. In this theory, OME is an active chronic bacterial disease rather than an aseptic inflammatory process. Experimental studies seem to confirm this theory because biofilm was found on the mucous membrane of the middle ear of chinchilla in otitis media experimentally induced by *Haemophilus influenzae* [62]. The mucosal biofilm model can explain the observation that metabolically active bacteria are present in negative cultures of OME exudates and that antibiotics are ineffective, while tympanostomy drainage is effective in the treatment of OME [63,64]. 

The unsatisfactory effect of OME antibiotic therapy may result not only from the genetic resistance of bacteria but also from the slowdown of their metabolism, independent of genetic conditions. The fact that antibiotics can make bacteria difficult to grow explains the postulate that antibiotic stress induces these pathogens to form biofilm [63,64]. 

Studies on the pharmacokinetics of orally administered antibiotics demonstrate that the killer-mediating drug concentration in vivo is readily available in the middle ear space. However, biofilm-forming bacteria are hundreds of times more resistant to antibiotics than planktonic bacteria, mainly due to the fact that mature parts of the biofilm grow slowly and therefore less frequently interact with the middle ear environment. Thus, the biofilm pattern can explain clinical observations that antibiotics are ineffective in the treatment of OME [63,64]. 

The biofilm hypothesis is also consistent with clinical observations that ventilation drainage is the most effective method of OME treatment. The non-ventilated middle ear is an ideal environment for biofilm formation because previous viral infections and persistent hypoxia disrupt the normal defense mechanisms of the mucous membrane. The healthy middle ear mucosa consists of ciliated epithelial cells that are involved in the bacterial cell purification mechanisms. It has been proven that the epithelium of the middle ear in OME is deprived of cilia, while rich in secretory cells (their number increases during OME). Placement of the tympanostomy tube restores ventilation of the middle ear and causes an increase in the partial pressure of oxygen, changing the biofilm phenotype. Suction of exudate breaks and reduces the mass of biofilm, increases the oxygen level, and leads to renewal of the ciliary epithelium [65].

However, the following limitations should be noted. Firstly, due to the age of the patients and the lack of cooperation between audiologists and patients younger than 7 years of age, the pure-tone threshold audiometry was possible only in half of the patients (53.95%). In the remaining children, only impedance audiometry and otoacoustic emissions were tested. In our next study we would like to include only patients older than 7 years to be able to determine hearing thresholds in all patients. Moreover, in our study there was no control group of healthy children without any otological problems; however, in future research we will include a control group and compare the microbiological profile between the study and the control group.

## 5. Conclusions

The obtained results allow the following conclusions to be drawn:

1. *Staphylococcus* spp. in OME may form biofilms, which can explain the chronic character of the disease and negative culture results.

2. Pathogenic bacteria typical of upper respiratory tract infections (*Haemophilus influenzae*, *Streptococcus pneumoniae* and *Staphylococcus aureus MSSA*) as well as opportunistic bacteria (*Staphylococus* spp., *Diphtheroids*, *Streptococcus viridans* and *Neisseria* spp.) may be involved in the etiopathogenesis of otitis media with effusion.

3. The degree of hearing loss correlated with the presence of bacteria, as evidenced by the results of microbiological tests. The degree of hearing loss was significantly higher in patients from which positive bacterial cultures were obtained. The difference in the audiometric test was on average 10 dB and was statistically significant (*p* = 0.0008).

Better understanding of the pathogens involved in otitis media with effusion development will help to identify high-risk patients and to explain the pathogenesis of the disease. This, in turn, will provide adequate opportunities for the design and implementation of diagnostic tests and effective therapeutic strategies for otitis media with effusion. Hearing can be monitored in patients with positive bacterial cultures and therefore a permanent hearing loss due to otitis media with effusion can be avoided.

## Figures and Tables

**Table 1:** Thermal profile of the first, outer amplification reaction (40 cycles from 2 to 4).

Type of Reaction	Time	Temperature
initial denaturation	2 min	95 °C
denaturation	30 s	95 °C
annealing step	60 s	58 °C
elongation	45 s	72 °C
final elongation	6 min	72 °C

**Table 2:** Thermal profile of the second, nested amplification reaction (40 cycles from 2 to 4).

Type of Reaction	Time	Temperature
initial denaturation	1 min	95 °C
denaturation	30 s	95 °C
annealing step	30 s	50 °C
elongation	30 s	72 °C
final elongation	6 min	72 °C

**Table 3:** Primer sequences, amplification conditions, and sizes of amplification products of *icaABCD* genes for *Staphylococcus epidermidis.*

PCR Product	Primer Sequences	Amplification Conditions	Product Size
*icaA*	Forward: 5′-GACCTCGAAGTC AATAGAGGT Reverse: 5′-CCCAGTATAACGTTGGATACC	60 s 94 °C 60 s 60 °C 2.5 min 72 °C	814 bp
*icaB*	Forward: 5′-ATGGCTTAAAGCACACGACGC Reverse: 5′-TATCGGCATCTGGTGTGACAG	60 s 94 °C 60 s 59 °C 2.5min 72 °C	526 bp
*icaC*	Forward: 5′-ATAAACTTGAATTAGTGTATT Reverse: 5′-ATATATAAAACTCTCTTAACA	60 s 94 °C 60 s 45 °C 2.5 min 72 °C	989 bp
*icaD*	Forward: 5′-AGGCAATATCCAACGGTAA Reverse: 5′-GTCACGACCTTTCTTATATT	60 s 94 °C 60 s 59 °C 2.5 min 72 °C	282 bp

**Table 4:** Degree of hearing impairment depending on the culture result.

Type of Bacteria	Mean Value-M	Standard Deviation-SD
Positive culture	35.9 dB	13.3 dB
Negative culture	25.9 dB	11.5 dB
t	5.659	
p	0.0008	
d	0.801	

t—standard error. p—calculated probability. d—effect size.

**Table 5:** Culture results.

Type of Bacteria	Percentage
Pathogenic	23.68
Opportunistic	76.32
Total	100.00

**Table 6:** Type and percentage of pathogenic bacteria in exudate collected from the middle ear space.

Type of Pathogenic Bacteria	Percentage
*Haemophilus influenzae*	38.89
*Streptococcus pneumoniae*	33.33
*Staphylococcus aureus MSSA*	27.78
Total	100.00

**Table 7:** Type and percentage of opportunistic bacteria in exudate collected from the middle ear space.

Type of Opportunistic Bacteria	Percentage
*Staphylococcus* spp.	74.14
*Diphtheroids*	20.69
*Streptococcus viridans*	3.45
*Neisseria* spp.	1.72
Total	100.00

**Table 8:** Degree of hearing impairment depending on the type of cultured bacteria.

Type of Bacteria	Mean Value-M	Standard Deviation-SD
Pathogenic bacteria	32.3 dB	12.0 dB
Opportunistic bacteria	27.5 dB	12.1 dB
t ^1^	3.025	
p ^2^	0.0192	
d ^3^	0.395	

^1^ t—standard error. ^2^ p—calculated probability. ^3^ d—effect size.

**Table 9:** Type and percentage of *Staphylococcus* spp.

Type of Bacteria	Percentage
*Staphylococcus epidermidis*	50.00
*Staphylococcus aureus*	18.75
*Staphylococcus sciuri*	12.50
*Staphylococcus capitis*	6.25
*Staphylococcus caprae*	6.25
*Staphylococcus verneri*	6.25

## Data Availability

The data presented in this study are available on request from the corresponding author. The data are not publicly available due to research participants’ privacy.

## References

[1] Ashoor A. (1994). Middle ear effusion in children: Review of recent literature. J. Fam. Community Med..

[2] Bennett K.E., Haggard M.P., Silva P.A., Stewart I.A. (2001). Behaviour and developmental effects of otitis media with effusion into the teens. Arch. Dis. Child..

[3] Rosenfeld R.M., Schwartz S.R., Pynnonen M.A., Tunkel D.E., Hussey H.M., Fichera J.S., Grimes A.M., Hackell J.M., Harrison M.F., Haskell H. (2013). Clinical Practice Guideline: Tympanostomy Tubes in Children. Otolaryngol. Head Neck Surg..

[4] Skoloudik L., Kalfert D., Valenta T., Chrobok V. (2018). Relation between adenoid size and otitis media with effusion. Eur. Ann. Otorhinolaryngol. Head Neck Dis..

[5] Rosenfeld R.M., Shin J.J., Schwartz S.R., Coggins R., Gagnon L., Hackell J.M., Hoelting D., Hunter L.L., Kummer A.W., Payne S.C. (2016). Clinical Practice Guideline: Otitis Media with Effusion (Update). Otolaryngol. Head Neck Surg..

[6] Elmagd E.A.A., Saleem T.H., Khalefa M.E., Elhawary B. (2019). Pathogenesis and Microbiology of Otitis Media with Effusion in Children. Int. J. Otolaryngol. Head Neck Surg..

[7] Chonmaitree T., Revai K., Grady J.J., Clos A., Patel J.A., Nair S., Fan J., Henrickson K.J. (2008). Viral Upper Respiratory Tract Infection and Otitis Media Complication in Young Children. Clin. Infect. Dis..

[8] Venekamp R.P., Burton M.J., van Dongen T.M., van der Heijden G.J., van Zon A., Schilder A.G. (2016). Antibiotics for otitis media with effusion in children. Cochrane Database Syst. Rev..

[9] Ahn J.H., Kang W.S., Kim J.H., Koh K.S., Yoon T.H. (2012). Clinical manifestation and risk factors of children with cleft palate receiving repeated ventilating tube insertions for treatment of recurrent otitis media with effusion. Acta Otolaryngol..

[10] Galletti B., Gazia F., Freni F., Nicita R.A., Bruno R., Galletti F. (2019). Chronic otitis media associated with cholesteatoma in a case of the say-barber-biesecker-young-simpson variant of ohdo syndrome. Am. J. Case Rep..

[11] Boonacker C.W.B., Rovers M.M., Browning G.G., Hoes A.W., Schilder A.G.M., Burton M.J. (2014). Adenoidectomy with or without grommets for children with otitis media: An individual patient data meta-analysis. Health Technol. Assess..

[12] Miura M.S., Mascaro M., Rosenfeld R.M. (2012). Association between otitis media and gastroesophageal reflux: A systematic review. Otolaryngol. Head Neck Surg..

[13] Straetemans M., Wiertsema S.P., Sanders E.A.M., Rijkers G.T., Graamans K., van der Baan B., Zielhuis G.A. (2005). Immunological status in the aetiology of recurrent otitis media with effusion: Serum immunoglobulin levels, functional mannose-binding lectin and Fc receptor polymorphisms for IgG. J. Clin. Immunol..

[14] Luong A., Roland P.S. (2008). The Link Between Allergic Rhinitis and Chronic Otitis Media with Effusion in Atopic Patients. Otolaryngol. Clin. North. Am..

[15] Kiris M., Muderris T., Kara T., Bercin S., Cankaya H., Sevil E. (2012). Prevalence and risk factors of otitis media with effusion in school children in Eastern Anatolia. Int. J. Pediatr. Otorhinolaryngol..

[16] Shishegar M., Faramarzi A., Kazemi T., Bayat A., Motamedifar M. (2011). Polymerase chain reaction, bacteriologic detection and antibiogram of bacteria isolated from otitis media with effusion in children, Shiraz, Iran. Iran. J. Med. Sci..

[17] Bluestone C.D., Stephenson J.S., Martin L.M. (1992). Ten-year review of otitis media pathogens. Pediatr. Infect. Dis. J..

[18] Greiner O., Day P.J.R., Altwegg M., Nadal D. (2003). Quantitative detection of Moraxella catarrhalis in nasopharyngeal secretions by real-time PCR. J. Clin. Microbiol..

[19] Karhdag T., Kutbettin D., Kaygusuz I., Ozden M., Yalcin S., Ozturk L. (2002). Resistant bacteria in the adenoid tissues of children with Otitis media with effusion. Int. J. Pediatr. Otorhinolaryngol..

[20] Miller M.B., Koltai P.J., Hetherington S.V. (1990). Bacterial antigens and neutrophil granule proteins in middle-ear effusions. Arch. Otolaryngol. Neck Surg..

[21] Murphy T.F., Kirkham C. (2002). Biofilm formation by nontypeable Haemophilus influenzae: Strain variability, outer membrane antigen expression and role of pili. BMC Microbiol..

[22] Poetker D.M., Lindstrom D.R., Edmiston C.E., Krepel C.J., Link T.R., Kerschner J.E. (2005). Microbiology of middle ear effusions from 292 patients undergoing tympanostomy tube placement for middle ear disease. Int. J. Pediatr. Otorhinolaryngol..

[23] Smith-Vaughan H., Byun R., Nadkarni M., Jacques N.A., Hunter N., Halpin S., Morris P.S., Leach A.J. (2006). Measuring nasal bacterial load and its association with otitis media. BMC Ear Nose Throat Disord..

[24] Stenfors L.E., Raisanen S. (1992). Occurrence of Streptococcus pneumoniae and Haemophilus influenzae in otitis media with effusion. Clin. Otolaryngol. Allied Sci..

[25] Ulualp S.O., Sahin D., Yilmaz N., Anadol V., Peker O., Gursan O. (1999). Increased adenoid mast cells in patients with otitis media with effusion. Int. J. Pediatr. Otorhinolaryngol..

[26] Aly B.H., Hamad M.S., Mohey M., Amen S. (2012). Polymerase Chain Reaction (PCR) Versus Bacterial Culture in Detection of Organisms in Otitis Media with Effusion (OME) in Children. Indian J. Otolaryngol. Head Neck Surg..

[27] Beswick A.J., Lawley B., Fraise A.P., Pahor A.L., Brown N.L. (1999). Detection of Alloiococcus otitis in mixed bacterial populations from middle-ear effusions of patients with otitis media. Lancet.

[28] Hendolin P.H., Kärkkäinen U., Himi T., Markkanen A., Ylikoski J. (1999). High incidence of Alloiococcus otitis in otitis media with effusion. Pediatr. Infect. Dis. J..

[29] Belfield K., Bayston R., Birchall J.P., Daniel M. (2015). Do orally administered antibiotics reach concentrations in the middle ear sufficient to eradicate planktonic and biofilm bacteria? A review. Int. J. Pediatr. Otorhinolaryngol..

[30] Davies D.G., Parsek M.R., Pearson J.P., Iglewski B.H., Costerton J.W., Greenberg E.P. (1998). The involvement of cell-to-cell signals in the development of a bacterial biofilm. Science.

[31] Dingman J.R., Rayner M.G., Mishra S., Zhang Y., Ehrlich M.D., Post J.C., Ehrlich G.D. (1998). Correlation between presence of viable bacteria and presence of endotoxin in middle-ear effusions. J. Clin. Microbiol..

[32] Ehrlich G.D., Veeh R., Wang X., William Costerton J., Hayes J.D., Hu F.Z., Daigle B.J., Ehrlich M.D., Christopher Post J. (2002). Mucosal biofilm formation on middle-ear mucosa in the chinchilla model of otitis media. J. Am. Med. Assoc..

[33] Erlandsen S.L., Kristich C.J., Dunny G.M., Wells C.L. (2004). High-resolution visualization of the microbial glycocalyx with low-voltage scanning electron microscopy: Dependence on cationic dyes. J. Histochem. Cytochem..

[34] Polachek A., Greenberg D., Lavi-Givon N., Broides A., Leiberman A., Dagan R., Leibovitz E. (2004). Relationship among peripheral leukocyte counts, etiologic agents and clinical manifestations in acute otitis media. Pediatr. Infect. Dis. J..

[35] Rayner M.G., Zhang Y., Gorry M.C., Chen Y., Post J.C., Ehrlich G.D. (1998). Evidence of bacterial metabolic activity in culture-negative otitis media with effusion. J. Am. Med. Assoc..

[36] Galli J., Ardito F., Calò L., Mancinelli L., Imperiali M., Parrilla C., Picciotti P.M., Fadda G. (2007). Recurrent upper airway infections and bacterial biofilms. J. Laryngol. Otol..

[37] Zuliani G., Carron M., Gurrola J., Coleman C., Haupert M., Berk R., Coticchia J. (2006). Identification of adenoid biofilms in chronic rhinosinusitis. Int. J. Pediatr. Otorhinolaryngol..

[38] Śliwa L., Hatzopoulos S., Kochanek K., Piłka A., Senderski A., Skarzyński P.H. (2011). A comparison of audiometric and objective methods in hearing screening of school children. A preliminary study. Int. J. Pediatr. Otorhinolaryngol..

[39] Kemp D.T. (2002). Otoacoustic emissions, their origin in cochlear function, and use. Br. Med. Bull..

[40] Musiek F.E., Shinn J., Chermak G.D., Bamiou D.E. (2017). Perspectives on the pure-tone audiogram. J. Am. Acad. Audiol..

[41] NHS (2019). Bacteriology: Catalase Test. UK Stand. Microbiol. Investig..

[42] Smole S.C., Aronson E., Durbin A., Brecher S.M., Arbeit R.D. (1998). Sensitivity and Specificity of an Improved Rapid Latex Agglutination Test for Identification of Methicillin-Sensitive and -Resistant Staphylococcus aureus Isolates. J. Clin. Microbiol..

[43] Chamberlain N. (2009). Coagulase Test for Staphylococcus Species. Am. Soc. Microbiol..

[44] Chapin K., Musgnug M. (2003). Evaluation of three rapid methods for the direct identification of Staphylococcus aureus from positive blood cultures. J. Clin. Microbiol..

[45] Pinto T.C.A., Souza A.R.V., De Pina S.E.C.M., Costa N.S., Neto A.A.B., Neves F.P.G., Merquior V.L.C., Dias C.A.G., Peralta J.M., Teixeira L.M. (2013). Phenotypic and molecular characterization of optochin-resistant streptococcus pneumoniae isolates from Brazil, with description of five novel mutations in the atpC gene. J. Clin. Microbiol..

[46] Arbique J.C., Poyart C., Trieu-Cuot P., Quesne G., Carvalho M.D.G.S., Steigerwalt A.G., Morey R.E., Jackson D., Davidson R.J., Facklam R.R. (2004). Accuracy of phenotypic and genotypic testing for identification of Streptococcus pneumoniae and description of Streptococcus pseudopneumoniae sp. nov. J. Clin. Microbiol..

[47] Gok U., Bulut Y., Keles E., Yalcin S., Doymaz M.Z. (2001). Bacteriological and PCR analysis of clinical material aspirated from otitis media with effusions. Int. J. Pediatr. Otorhinolaryngol..

[48] Lee P.Y., Costumbrado J., Hsu C.Y., Kim Y.H. (2012). Agarose gel electrophoresis for the separation of DNA fragments. J. Vis. Exp..

[49] Hassan A., Usman J., Kaleem F., Omair M., Khalid A., Iqbal M. (2011). Evaluation of different detection methods of biofilm formation in the clinical isolates. Brazilian J. Infect. Dis..

[50] Ziebuhr W., Krimmer V., Rachid S., Lößner I., Götz F., Hacker J. (1999). A novel mechanism of phase variation of virulence in Staphylococcus epidermidis: Evidence for control of the polysaccharide intercellular adhesin synthesis by alternating insertion and excision of the insertion sequence element IS256. Mol. Microbiol..

[51] De Silva G.D.I., Kantzanou M., Justice A., Massey R.C., Wilkinson A.R., Day N.P.J., Peacock S.J. (2002). The ica operon and biofilm production in coagulase-negative staphylococci associated with carriage and disease in a neonatal intensive care unit. J. Clin. Microbiol..

[52] Park C.-W., Han J.-H., Jeong J.-H., Cho S.-H., Kang M.-J., Tae K., Lee S.-H. (2004). Detection rates of bacteria in chronic otitis media with effusion in children. J. Korean Med. Sci..

[53] Galletti F., Cammaroto G., Galletti B., Quartuccio N., Di Mauro F., Baldari S. (2015). Technetium-99m (99mTc)-labelled sulesomab in the management of malignant external otitis: Is there any role?. Eur. Arch. Oto Rhino Laryngol..

[54] Black S., Shinefield H., Fireman B., Lewis E., Ray P., Hansen J.R., Elvin L., Ensor K.M., Hackell J., Siber G. (2000). Efficacy, safety and immunogenicity of heptavalent pneumococcal conjugate vaccine in children. Northern California Kaiser Permanente Vaccine Study Center Group. Pediatr. Infect. Dis. J..

[55] Caspary H., Welch J.C., Lawson L., Darrow D., Buescher S., Shahab S., Derkay C.S. (2004). Impact of pneumococcal polysaccharide vaccine (Prevnar) on middle ear fluid in children undergoing tympanostomy tube insertion. Laryngoscope.

[56] Palmu A.I., Saukkoriipi P.A., Lahdenkari M.I., Kuisma L.K., Makela P.H., Kilpi T.M., Leinonen M. (2004). Does the presence of pneumococcal DNA in middle-ear fluid indicate pneumococcal etiology in acute otitis media?. J. Infect. Dis..

[57] Choi Y.C., Park Y.S., Yeo S.W., Chae S., Jung D.G., Kim S.W. (1998). Detection of Haemophilus influenzae and Streptococcus pneumoniae by polymerase chain reaction (PCR) in chronic Otitis media with effusion (OME). Korean J. Otolaryngol..

[58] Hall-Stoodley L., Hu F.Z., Gieseke A., Nistico L., Nguyen D., Hayes J., Forbes M., Greenberg D.P., Dice B., Burrows A. (2006). Direct detection of bacterial biofilms on the middle-ear mucosa of children with chronic otitis media. J. Am. Med. Assoc..

[59] Stenqvist M., Anniko M., Pettersson Å. (1997). Effect of Pseudomonas aeruginosa exotocin A on inner ear function. Acta Otolaryngol..

[60] Büttner H., Mack D., Rohde H. (2015). Structural basis of Staphylococcus epidermidis biofilm formation: Mechanisms and molecular interactions. Front. Cell. Infect. Microbiol..

[61] Daniel M., Imtiaz-Umer S., Fergie N., Birchall J.P., Bayston R. (2012). Bacterial involvement in Otitis media with effusion. Int. J. Pediatr. Otorhinolaryngol..

[62] Suzuki K., Bakaletz L.O. (1994). Synergistic effect of adenovirus type 1 and nontypeable Haemophilus influenzae in a chinchilla model of experimental otitis media. Infect. Immun..

[63] Fergie N., Bayston R., Pearson J.P., Birchall J.P. (2004). Is otitis media with effusion a biofilm infection?. Clin. Otolaryngol. Allied Sci..

[64] Qureishi A., Lee Y., Belfield K., Birchall J.P., Daniel M. (2014). Update on otitis media—Prevention and treatment. Infect. Drug Resist..

[65] Rosenfeld R. (1995). Nonsurgical management of surgical otitis media with effusion. J. Laryngol. Otol..

